# Phonemic awareness of English second language learners

**DOI:** 10.4102/sajcd.v64i1.164

**Published:** 2017-01-30

**Authors:** Maria le Roux, Salome Geertsema, Heila Jordaan, Danie Prinsloo

**Affiliations:** 1 Department of Speech-Language Pathology and Audiology, University of Pretoria, South Africa; 2 Department of Speech Pathology and Audiology, University of the Witwatersrand, South Africa; 3 Department of African Languages, University of Pretoria, South Africa

## Abstract

**Background:**

The PA skills of phonological blending and segmentation and auditory word discrimination relate directly to literacy and may be weak in English second language (EL2) learners. In South Africa, literacy skills have been found to be poor in especially EL2 learners.

**Objectives:**

The purpose of this paper is to determine the effects of vowel perception and production intervention on phonemic awareness (PA) and literacy skills of Setswana first language (L1) learners. These learners are English second language (EL2) learners in Grade 3.

**Method:**

The present study employed a quasi-experimental, pre-test–post-test design.

**Results:**

The findings of low–literacy skill levels concurred with previous investigations. However, post-test results of intervention in PA seemed to improve the literacy skills of EL2 learners.

**Conclusion:**

PA skills should be a crucial part of the literacy curriculum in South Africa.

## Introduction

Phonemic awareness (PA), as a specific sub-skill of phonological awareness, is the refined ability to segment words into constituting sounds and blend these sounds to form new words (Yeong & Rickard Liow, 2012). This type of awareness is the more ‘sophisticated’ of PA skills according to Stewart (2004, p. 32), requiring recognition of the phoneme as the smallest unit of language that can bring about a difference in meaning.

PA skills develop during the pre-school years and during the beginning stages of learning to read (Adams, 1990). Children in Grades RR, R and 1 should understand that words are constructed by blending phonemes together, for example, ‘cat’ is formed by blending /k/, /æ/ and /t/. Simultaneously, the word ‘cat’ can be segmented into /k/, /æ/ and /t/. Learners should also be able to recognise that a difference in meaning occurs when the /k/ in ‘cat’ is replaced with /m/ and a new word, ‘mat’, is formed.

When a child learns that a phoneme contributes to distinguishing meaning in words, PA is triggered. Well-developed PA skills enable the child to manipulate phonemes by blending, segmenting or replacing individual phonemes in words to generate new words (Cisero & Royer, 1995). These skills will assist in mastering phonic skills such as phoneme–grapheme coupling required for reading and spelling.

When considering the English language of learning and teaching (ELoLT) context in South Africa, the question as to how EL2 learners develop PA skills (and phonological awareness skills in general) arises. Research posits that those PA skills already developed in the acquisition of the first language (L1), including knowledge of the phonological system of the L1, will be transferred to the second language (L2) (Anderson, 2004).

The success with which a child is able to make such transfers depends not only on the level of PA skills but also on the linguistic characteristics of the two languages in question. PA skills development seems to be parallel when comparing most of the alphabetic languages, while the rate of development and level of aptitude vary (Anthony & Francis, 2005; Ziegler & Goswami, 2005). Yeong and Rickard Liow (2012) posited that this variation is because of the dissimilarities in the characteristics of the children’s oral language. The less complicated the phonological structure of the language, the less sensitive to PA the child seems to be (McBride-Chang et al., 2008; Melhuish et al., 2008; Yeong & Rickard Liow, 2012).

This variation of PA skills development could apply to Setswana L1 learners in the ELoLT environment. Setswana, in contrast to English, has a smaller vowel system, consisting of seven basic vowels, four raised vowels and no diphthongs (Cole, 1955). In contrast, English distinguishes 19 vowel sounds (Bekker, 2009). Additionally, while English distinguishes long monophthongs, Setswana does not. Therefore, Setswana L1 learners often do not distinguish the long English monophthongs. This means that the Setswana L1 learner has developed less advanced PA skills to discriminate between the vowels and the diphthongs of ELoLT (Seeff-Gabriel, 2003).

Lack of sufficiently developed PA skills is concerning as these skills are regarded as the best predictor for reading ability during the early school years (Cockcroft & Alloway, 2012; Phillips, Clancey-Menchetti & Lonigan, 2008). Of these, segmentation and blending are the most important ‘sub-skills’ required when a child starts to read (Yeong & Rickard Liow, 2012). A sufficient level of these skills will enable the young learner to decode words during the initial stages of literacy (Koda, 2007).

Research on the transition to ELoLT in Grade 4 in South Africa indicated that the PA skills of EL2 learners were extremely low (Pretorius, 2014). This has a negative influence on the literacy skills and academic performance of these young learners as decoding skills predict reading comprehension, accuracy and fluency in the beginning stages of reading in both the L1 and L2 (Van Staden, 2011). The results of the pre-Progress in International Reading Literacy Study 2011 (prePirls) indicated that Grade 4 EL2 learners scored 80 points lower than their EL1 peers (Howie, Van Staden, Tshele, Dowse & Zimmerman, 2012). The difference in scores becomes meaningful when Howie et al. (2012) explain that a difference of 80 points relates to a difference of about 2 years less schooling for the EL2 group. In addition to the prePirls2011 (2012) results, the Annual National Assessment indicated that in 2014 only 52% of Grade 3 learners passed the literacy assessment (Department of Basic Education, 2015).

With research indicating that learners with low levels of literacy often do not have sufficient PA skills, the aim of this study was to assess the effects of vowel perception and production intervention on PA and literacy skills of EL2 (Setswana L1 speaking) learners in Grade 3.

## Research design

A quasi-experimental design was employed. We aimed to rule out the possible effects of non-intervention variables. The variables controlled for are *testing* (pre-test–post-test design), *maturation* [period between pre-test and post-test was minimised to eliminate biological changes (12 weeks)] (Armstrong & Van Mechelen, 2008), *instrumentation* (standardised tests were used for assessment), *regression* (the mean of results of participants were used; not individual scores), *mortality* and/or *attrition* (intervention was made stimulating through the use of feedback in the form of real-time spectrograms and intermittent positive reinforcement – verbal and tangible – by the student therapists . This was done to prevent drop-out) and *selection* (participants in groups were as similar as possible). Simultaneously, the study used a comparative design.

### Participants

English second language (ESL) speaking participants were randomly assigned to the EL2 and control group (CG) groups. Participants of the EL1 group were also randomly selected.

#### EL1 group

Twelve 8- to 10-year-old South African English L1 speakers, attending an English medium school in Pretoria, were selected for the comparative pre- and post-tests. Bekker (2009) stated that this variety of English can be seen as standard South African English. This group was seen as the ‘norm’ and did not receive intervention.

#### EL2 group

Fifteen 8- to 10-year-old EL2 (Setswana L1 speaking) learners, attending two English medium primary schools in the Moot (Pretoria), were selected for comparative pre- and post-tests. These participants received additional input concerning the English vowel system by final-year EL1 Speech-Language Therapy (SLT) students.

#### Control group

Fifteen 8–10-year-old EL2 (Setswana L1 speaking) learners were selected. These participants did not receive intervention. They were selected from the same schools as participants in the EL2 group.

### Participant characteristics

#### Age

Participants were in Grade 3 and aged between 8 and 10 years old. These learners were exposed to ELoLT for 3–4 years, and basic literacy skills should have been established at this stage.

#### Gender

Participants were male and female learners. At least 15 Setswana L1-speaking learners were needed in each group; however, there were insufficient male-only or female-only learners available for selection at the primary schools that were willing to participate.

#### Socio-economic and geographical status

Participants attended former model-C primary schools. The EL1 group school is in a higher socio-economic area. Although the EL2 and CG groups’ schools are in lower socio-economic areas, these participants were not necessarily from lower socio-economic households. School records showed many parents find it logistically easier to enrol the children in those particular schools.

#### Speech and hearing abilities

Participants displayed normal speech and hearing abilities as screened by the student SLTs and teachers.

## Materials

All materials are standardised, but not necessarily for the South African ESL population. As such, these tests are interpreted as only an indication of the level of EL2 functioning.

### The test of auditory processing skills

Test of auditory processing skills, *Third edition* *(TAPS-3*) (Martin & Brownell, 2005), was employed to investigate the auditory processing of the participants. The first three sub-tests (phonological processing) were the focus of the research. The sub-tests evaluate *Word Discrimination*, *Phonological Segmentation*, and *Phonological Blending*. Not only does this test assess the phonemic skills of blending and segmentation but also it focuses on the discrimination of phonological similarities and differences in word pairs. As word discrimination plays an important role in especially the L2 class room, it was necessary to determine the participants’ skills on this sub-test.

### The one-minute reading test

The *One-Minute Reading Test* (Transvaal Education Department, 1987) was used to assess the reading accuracy skills. This test consists of monosyllabic words and the reading is assessed against time to produce a chronological reading age.

### The UCT Spelling Test

The *UCT Spelling Test* (University of Cape Town, 1985) was used to assess the spelling skills. This test is a standardised South African spelling test containing stimulus words of increasing complexity.

## Procedures

### Procedure for data collection

#### Assessment protocol

Participants were assessed in a venue at the schools by female English L1-speaking SLT students who were trained to conduct the tests. The order of the tests was randomised to counterbalance the effects of fatigue on the last administered test. Testing took approximately 60 min per participant. The results were calculated and captured in Microsoft Excel (2010). After 12 weeks of intervention with the EL2 group, the participants were re-assessed using the same protocol.

#### Intervention protocol

For the vowel perception and production intervention, the researchers combined the Traditional Articulation Approach (Van Riper & Emerick, 1984) and Cycles Phonological Remediation Approach (Hodson, 2006) (Appendix). Aspects from the Traditional Articulation Approach followed a specific course beginning with sensory-perceptual training (ear training – identification, isolation, stimulation, and discrimination of vowel contrasts). Production training followed in which the target vowel sound was established or acquired and then stabilised (Creaghead, Newman & Secord, 1989). To stabilise the sounds, participants practiced the production of the vowel sounds in isolation, nonsense syllables and words. Adapted aspects of the Cycles Phonological Remediation Approach were also used to guide intervention. The nonsense syllable within each repetition of this section of the session was repeated with increasing complexity. This pattern formed part of the cycle as in the adapted shorter version of the Cycles approach of Hodson (2006). An example is nana [nænæ] – nanu [nænƱ] in a single session; revision of these stimuli in the following session and addition of another vowel and or syllable, such as nana [nænæ] – nanu [nænƱ] – nani [nænƱ] or nana [nænæ] – nananu [nænænƱ].

Because intervention was only given once a week, revision was offered to be in line with educational approaches. Target sounds were reviewed with production practice of words from the previous session. A listening activity and production practice followed. The sessions incorporated segmentation and blending activities because research indicates that this is the way in which PA skills should be taught to enable reading and spelling ability (Moats, 2007). These activities emulate the approach that teachers (may) use to teach literacy skills, which could enhance the benefits of the intervention. The words for reading and spelling practice were selected using age-appropriate reading lists as suggested by the Curriculum Assessment Policy Statement (Department of Basic Education, 2011). Ten words for spelling and reading were randomly selected for each vowel where possible. The words presented in the sessions were not contained in the *UCT Spelling Test* or the *One-Minute Reading Test* used for assessing the participants.

The researchers targeted each of the 19 South African English (Bekker, 2009) vowel sounds with a maximum of two vowel sounds covered per session. Twelve weekly intervention sessions were conducted. During every second intervention session, a single vowel sound was targeted. The intervention session concluded with revision of the vowels previously addressed. When no revision was conducted, two vowel sounds were targeted.

Each session lasted 45 min. Sessions were conducted in a group setting with three participants in each session and one student SLT. When two vowel sounds were targeted the student SLTs would introduce the first vowel sound and perform the discrimination, production and reading task. The second vowel sound would be introduced and targeted. The vowel sounds were then targeted concurrently for the spelling task. The intervention programme followed a set structure for each session. During sessions, the student SLTs gave augmented verbal feedback about the different auditory and articulatory characteristics of the relevant vowel(s).

### Procedure for data analysis

Results on the sub-tests of *TAPS-3* were compared pre- and post-intervention and are presented in tables and bar graphs. The data on the reading and spelling assessments were also analysed and compared pre- and post-intervention. Data were analysed using Stata 12.1 (Stata Corporation, College Station, TX, USA). Descriptive statistics were calculated for all variables, viz. mean and standard deviation. Additionally, the median and interquartile ranges were determined for the highly skewed pre-test scores for spelling and phonological blending. Student’s paired *t*-tests were used to test for significant differences between the pre- and post-test results of normally distributed variables within each of the participating groups. Likewise, the Wilcoxon signed-rank test was used in the analysis of non-normal variables. One-way and Kruskal–Wallis analysis of variance (ANOVA) identified differences between the means of each variable, across all groups. Where a significant difference in mean scores was observed, further post hoc analyses were carried out to determine in which groups the differences occurred. Differences with *p* < 0.05 were deemed statistically significant.

## Ethical considerations

Permission for the study was obtained from the University of Pretoria, the Gauteng Department of Education, and the participating schools. Parental consent and participant assent were obtained. During assessments and intervention, care was taken to minimise any discomfort that the participants might have experienced. Participants were praised for their diligence and willingness to participate. None of the participants withdrew from the project.

The CG group did not receive intervention. However, the researchers offered the option of intervention after completion of the present research to the parents and participants of this group.

## Results and discussion

### Performance on the Word Discrimination sub-test of the *TAPS-3*

**FIGURE 1:**
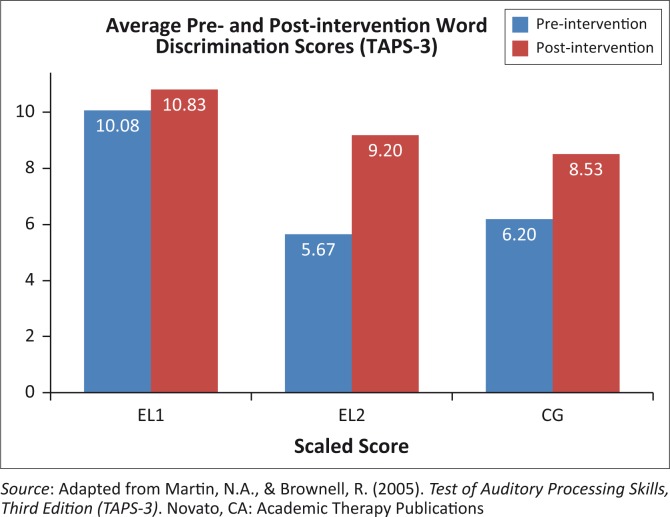
Average scaled scores of word discrimination of all participants across all groups.

The average scores on the word discrimination sub-test are illustrated in Figure 1.

In Tables 1 and 2, the results of the *t*-tests for significant differences within and between groups on the word discrimination sub-test are provided.

**TABLE 1:** Results of *t*-tests for significant differences in pre- and post-intervention word discrimination scores *within* groups.

Groups	Pre-tx	Post-tx
	*t*-value	*p*
EL2	-3.11	0.01†
CG	-3.70	0.002†
EL1	-1.09	0.30

† Statistically significant.

**TABLE 2:** Results of *t*-tests for significant differences *between* groups on word discrimination scores.

Groups	Pre-tx		Post-tx	
	*t*-value	*p*	t-value	*p*
EL1 and EL2	3.76	< 0.001†	1.63	0.12
EL1 and CG	3.53	0.002†	2.65	0.01†
CG and EL2	−0.50	0.62	0.73	0.47

† Statistically significant.

The paired *t*-test confirmed a significant difference on the word discrimination sub-test between pre- and post-intervention results in the EL2 (*p* = 0.01) and CG (*p* = 0.002) groups. These groups improved from an average of 5.67 to 9.2 and 6.2 to 8.53, respectively. The EL2 group showed an improvement of 62.4% compared to the 37.6% of the CG, indicating that the intervention was effective in improving word discrimination skills. The CG group also showed a significant difference in their pre- and post-intervention scores, although it was not enough to bring them to the level of the EL1 group. These results provide further support for the positive influence of the intervention; that is, maturation alone is not enough to close the gap between these two groups.

The difference between the EL2 and CG groups was not significant pre-intervention (*p* = 0.62) but both the EL2 (*p* < 0.001) and CG groups (*p* = 0.002) scored significantly below the EL1 group. Although the CG improved significantly from pre- to post-intervention (Table 2), its mean score remained significantly different from the EL1 group (*p* = 0.01) after intervention, unlike the EL2 group, who no longer scored significantly below the EL1 group (*p* = 0.12). These results would indicate that the EL2 group improved significantly on this measure (5.67–9.20 post-intervention), further confirming the effectiveness of the intervention.

### Performance on the phonological segmentation sub-test of the *TAPS-3*

**FIGURE 2:**
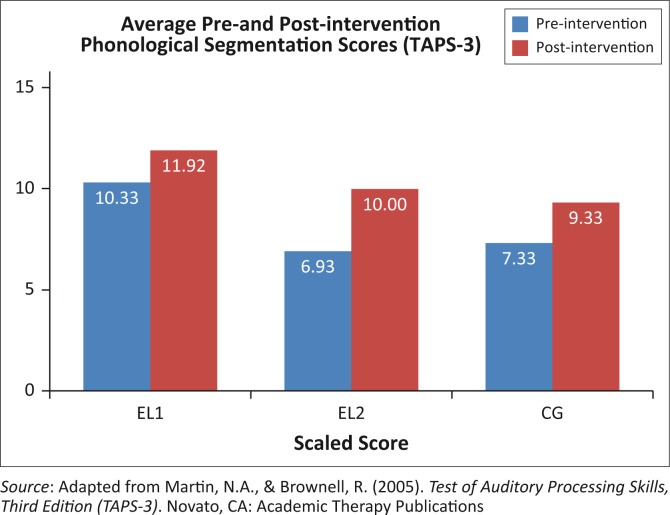
Average scaled scores on phonological segmentation across all groups.

The average scores on the phonological segmentation sub-test of the *TAPS-3* are illustrated in Figure 2.

Statistically significant differences between the group means were evident in the pre-intervention scores (*p* = 0.04) but not in the post-intervention (*p* = 0.08) scores. The statistical within-group comparison between pre- and post-intervention scores is provided in Table 3.

**TABLE 3:** Results of *t*-tests for significant differences in pre- and post-intervention scores on phonological segmentation scores *within* groups.

Groups	Pre-tx	Post-tx
	*t*-value	*p*
EL2	-3.88	0.002†
CG	-3.09	0.01†
EL1	-2.37	0.04†

† Statistically significant.

The paired *t*-test confirmed significant improvements from pre- to post-intervention phonological segmentation skills in all three groups (EL1 *p* = 0.04, EL2 *p* = 0.002, CG *p* = 0.01). Table 4 portrays the results of *t*-tests for significant differences between groups.

**TABLE 4:** Results of *t*-tests for significant differences *between* groups on Phonological Segmentation scores.

Groups	Pre-tx		Post-tx	
	*t*-value	*p*	*t*-value	*p*
EL1 and EL2	2.58	0.02†	1.72	0.10
EL1 and CG	2.18	0.04†	2.35	0.03†
CG and EL2	-0.30	0.77	0.60	0.56

† Statistically significant.

Post-intervention scores in the EL1 and EL2 groups were not significantly different. This would indicate that the EL2 group improved significantly in this measurement, to the extent that no statistically significant difference between them and the EL1 group could be observed. This improvement in EL1 group post-intervention scores suggests that the intervention was effective.

### Performance on the Phonological Blending sub-test of the *TAPS-3*

**FIGURE 3:**
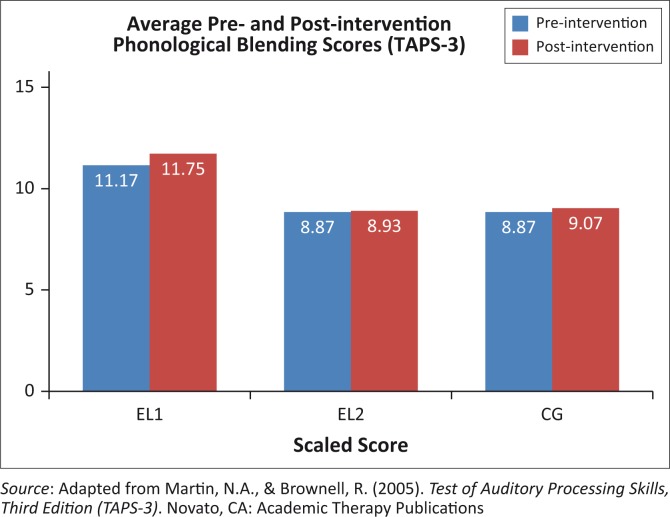
Average scaled scores of phonological blending of all participants across all groups.

The average scores on the phonological blending sub-test of the *TAPS-3* are illustrated in Figure 3.

The statistical within-group comparisons between pre- and post-intervention scores are provided in Table 5.

**TABLE 5:** Results of *t*-tests for significant differences in pre- and post-intervention phonological blending scores *within* groups.

Groups	Pre-tx	Post-tx
	*t*-value	*p*
EL2	-0.11	0.91
CG	-0.25	0.81
EL1	-0.76	0.46

† Statistically significant.

Wilcoxon signed-rank tests confirmed that there was no significant improvement between the pre- and post-intervention scores within any of three groups, including the EL2 group. Therefore, the intervention did not have an effect on phonological blending skills. Table 6 portrays the results of *t*-tests for significant differences between groups.

**TABLE 6:** Results of *t*-tests for significant differences in phonological blending scores *between* groups.

Groups	Pre-tx		Post-tx	
	*t*-value	*p*	*t*-value	*p*
EL1 and EL2	2.10	0.05	3.15	0.004†
EL1 and CG	1.61	0.12	2.62	0.02†
CG and EL2	-0.69	0.49	-0.14	0.89

† Statistically significant.

Differences between the pre-intervention scores of the EL1 and EL2 groups (*p* = 0.05) and the EL1 and the CG (*p* = 0.12) were not significant, implying that the groups had similar levels of phonological blending ability at the start of the study. Post-intervention results indicate that significant differences exist between the EL2 and EL1 groups (*p* = 0.004) as well as between the CG and EL1 groups (*p* = 0.02). These differences may be because of an improvement in the EL1 group scores from 11.17 to 11.75 (Figure 3).

### Performance on the reading skills scores

**FIGURE 4:**
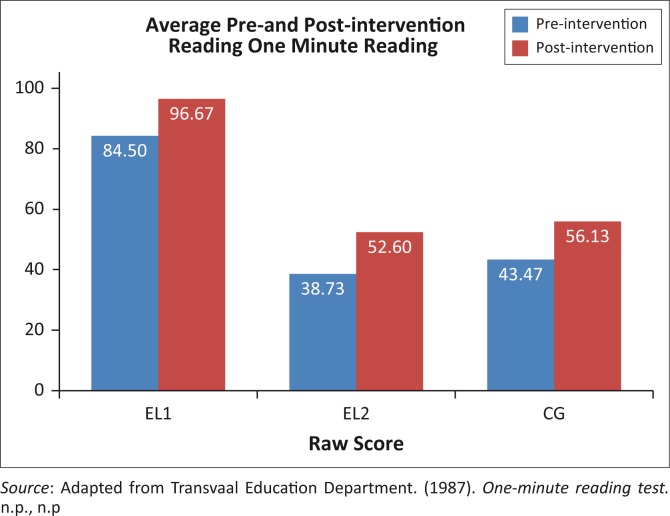
Average scaled scores of reading accuracy of all participants across all groups.

The average scores for reading skills are illustrated in Figure 4. The within- and between-group comparisons follow directly thereafter.

The statistical comparison between pre- and post-intervention scores on the reading assessment *within* all three groups is provided in Table 7.

**TABLE 7:** Results of *t*-tests for significant differences in pre- and post-intervention scores on reading accuracy assessment *within* groups.

Groups	Pre-tx	Post-tx
	*t*-value	*p*
EL2	-8.07	< 0.001†
CG	-4.95	< 0.001†
EL1	-2.58	0.03†

† Statistically significant.

The paired *t*-test confirmed significant differences between the pre- and post-intervention reading results in all three groups. All three groups’ reading skills could be seen to have improved. However, the EL2 group improved the most, with 35.8% compared to the 29.1% improvement of the CG and 14.1% improvement of the EL1 group. The EL2 group thus showed significant improvement in reading abilities (38.73–52.6 post-intervention), which could be attributed to the intervention received. Table 8 portrays the between-group comparisons.

**TABLE 8:** Results of *t*-tests for significant differences in reading scores *between* groups.

Groups	Pre-tx		Post-tx	
	*t*-value	*p*	t-value	*p*
EL1 and EL2	4.69	< 0.001†	4.94	< 0.001†
EL1 and CG	4.63	< 0.001†	4.93	< 0.001†
CG and EL2	-0.54	0.59	-0.39	0.70

† Statistically significant.

**TABLE 9:** Results of *t*-tests for significant differences in pre- and post-intervention scores on spelling *within* groups.

Groups	Pre-tx Post-tx	
	*z*‡	*p*
EL2	-3.37	0.001†
CG	-3.40	0.001†
EL1	-3.03	0.003†

† Statistically significant;

‡ Wilcoxon signed-rank test.

Both the EL2 and CG groups performed significantly below the EL1 group in the pre- and post-intervention assessments. No significant difference between CG and EL2 reading accuracy scores were observed.

### Performance on the spelling skills scores

**FIGURE 5:**
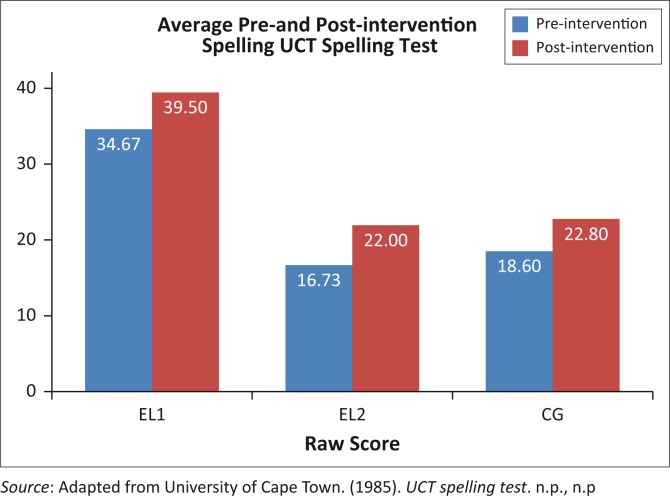
Average scaled pre- and post-intervention scores of all groups on the UCT Spelling Test.

The average scores for spelling skills are illustrated in Figure 5. The within- and between-group comparisons follow directly thereafter.

Wilcoxon signed-rank tests confirmed a significant difference in pre- versus post-intervention results for all three groups. All three groups’ reading skills could be seen to have improved. However, when analysing the spelling scores, the EL2 group once again shows a significant improvement of 31.5% compared to the 22.6% improvement of the CG and 13.9% improvement of the EL1. Note that this improvement within the EL1 group was also significant. However, even after the intervention, the spelling skills of the EL2 group, as measured by the UCT Spelling Test, are still 44% lower than those of the EL1 group. Table 10 displays the significant differences in spelling scores between groups.

**TABLE 10:** Results for significant differences in spelling scores *between* groups.

Groups	Pre-tx		Post-tx	
	*z*‡	*p*	*t*-value	*p*
EL1 and EL2	3.47	< 0.001†	4.09	0.004†
EL1 and CG	3.77	< 0.001†	4.69	< 0.001†
CG and EL2	-1.02	0.31	-0.21	0.83

† Statistically significant;

‡ Wilcoxon signed-rank test.

Significant differences between groups were observed between the pre- and post-intervention spelling scores of EL1, EL2 and CG groups. These findings underline the similarity of the EL2 and CG to each other, and the significant difference in their results compared to the results of the EL1 group. The results were to be expected as the EL2 and CG comprised EL2 learners, while the EL1 group consisted of EL1 learners.

## Discussion

The *word discrimination* sub-test determined whether the participants could recognise phonological differences and similarities within word pairs (Martin & Brownell, 2005). The participants had to indicate whether the members of the word pairs were the same or different (e.g. in the word pair ‘miss: mess’). Eleven of the 20 word pairs consisted of words in which the vowels differ. Because it has been established that L2 speakers find it difficult to distinguish all the vowels and diphthongs of English (Seeff-Gabriel, 2003), the low word discrimination scores of the L2 learners could be attributed to this. Another possible reason for the discrepancy in scores between the EL1 and EL2 groups is proposed by Phillips et al. (2008): word discrimination skills are related to word meaning. Therefore, it is possible that the EL2 participants struggled to recognise the meaning of the words presented and could not discriminate between the words because of meaning loss that occurred. Therefore, increasing vocabulary knowledge as part of oral skills is of utmost importance in the ELoLT environment (Phillips et al., 2008). Lonigan (2007) explained that the more enriched the vocabulary of a learner becomes, the more minimal pairs with distinctive sounds will form part of the lexicon of the learner. Such an increase will enhance phonological and PA abilities. The significant increase in post-intervention scores of the EL2 group indicated that additional input such as that described in the intervention plan may improve the ability of the EL2 learner to discriminate between sounds in minimal word pairs. These increased scores also suggest that the EL2 group became more aware of the different vowels of English because more than 50% of the word pairs differed in the vowel.

The *phonological segmentation* tasks of the *TAPS-3* not only focus on syllable deletion but also on the deletion of phonemes in various positions. It is a sophisticated skill that develops from, or at least concurrently with, print experiences during the beginning reading phase (Cisero & Royer, 1995). Young learners struggle to realise that words are constructed by different phonemes when they do not have adequate print experience (Melhuish et al., 2008). In South Africa, many EL2 learners are from a print-deprived environment (Howie et al., 2012). This could be cited as a reason for the fact that the EL1 participants achieved significantly higher pre-intervention scores than their EL2 peers. The increase in post-intervention scores of the EL2 group indicates that the input received by this group improved their segmentation skills considerably. The intervention seems to have increased the awareness of the sounds of English in the EL2 group, not only of the vowels but also of the consonants.

The *phonological blending* sub-test requires the participant to blend the phonemes produced in isolation to form the required word. Blending tasks are deemed easier than segmentation tasks (Phillips et al., 2008) and the overall high scores of all three groups (compared to those of the segmentation tasks) confirmed this assumption. This test is the only sub-test of the *TAPS-3* in which the EL2 group did not improve their scores post-intervention. A disturbing occurrence is that, although there was no significant difference between the scores of the EL1 and the EL2 groups before intervention, a significant difference between these groups was noted after intervention because of improvement of the phonological blending skills of the EL1 learners. Because phonological blending is considered to be easier than phonological segmentation, the gap developing between the EL1 and EL2 groups is a cause for concern.

The results obtained from the two *literacy skills* assessments confirm the direct impact of PA on these skills. The literacy skills of the EL2 participants measured below the level of their EL1 peers. Although an improvement in the abilities of the EL2 group can be noted after intervention, the skills levels were still low. An improvement was not sufficient for the EL2 group to ‘catch up’ with the EL1 group. This may be because of the fact that intervention took place only once a week for 45 min, instead of additional input being given throughout the day, as suggested by researchers like Trehearne, Healy, Cantalini-Williams and Moore (2004).

The age at which the L2 learner receives additional input to enhance his and/or her phonological awareness skills and phonological knowledge of the LoLT may play an important role in the poor EL2 literacy skills as well. While in this study the intervention was given in Grade 3, the ideal time for structured literacy instruction is considered to be pre-school (Lessing & De Witt, 2005). Because improvement in the literacy skills of the EL2 group can be noted, it is suggested that much more input concerning PA skills as well as oral language skills should be given to EL2 learners in Grade R already.

## Conclusion

Sufficiently developed PA skills, especially segmentation and blending, are seen as the best predictor for successful literacy acquisition during early school years (Cockcroft & Alloway, 2012; Moats, 2007). PA seems to be most at risk for EL2 learners because of differences in the phonetic repertoire of the respective languages. Children who have a first language with a less complicated phonological structure, such as that of Setswana, may have less sensitive PA skills (McBride-Chang et al., 2008; Yeong & Liow, 2012). The results of the present investigation also support this theoretical hypothesis. Our EL2 participants displayed significant challenges not only in the PA domains, particularly in word discrimination and phoneme segmentation, but also in phonological blending. These challenges have a negative impact on their decoding (reading) skills and encoding (spelling) skills. Not only is there limited time available to address literacy instruction (Department of Basic Education, 2011) but also it seems from our study that the even more important building blocks of these literacy skills, and the additional challenges posed by second language learning, may also be lacking and prohibiting these children from effectively attaining literacy. Therapists and educationalists should take note of the possible lack of sufficient PA skills in especially EL2 learners. Therefore, intensive PA training should be introduced into the curriculum as early as possible. This should enable the EL2 learner to become familiar with the phonological system of English, which in turn may lead to more successful literacy acquisition.

## Limitations and recommendations for future research

A few limitations should be noted. The South African spelling and reading tests used are relatively dated. Only two small peer-matched EL2 groups of participants were available for this study. These two groups came from middle-class former model-C schools.

Additional comparisons should be drawn between different socio-economic groups, provinces and other languages to direct educators towards an intervention program. Another recommendation for future research is to explore the underlying assumption that phonological awareness of consonants should be less problematic for Setswana EL2 learners. This is because the largest difference in phoneme inventories between these two languages occurs on the level of vowel systems. In addition, testing for language difference versus language disorder would be advisable. Such testing would be useful to compare the semantic knowledge of the groups because vocabulary contributes to PA and literacy skills as well. Investigations into the association of socio-economic status with limited early literacy experiences and vocabulary in EL2, for example, should be conducted. A final recommendation would be to utilise inter-rater reliability measures to compare the student SLTs assessment and intervention sessions.

## Appendix 1

**TABLE 1-A1:** Intervention hierarchy.

Minutes	Category	Instructions given
1–5	Introduction of sound and identification	Today’s sound is (produce sound). When I say the sound, my tongue does this (describe sound in terms of vowel production dimensions). When we write the sound it looks like this (give all possible written representations of sound).
6–12	Auditory discrimination	I want you to listen carefully. I am going to say 10 sounds. I want you to listen out for this sound (produce target sound). Whenever you hear it, I want you to clap your hands.
13–19	Production training: Isolation	I am going to say a sound and I want you to listen carefully (produce sound). Look at the computer. This is what my voice looks like when I say the sound. I am going to say it again, and then you can see what it looks like. I would like you to try and when you get it right, it will look like mine at the top. Now you try. (Participant attempts production).
20–27	Production training: Nonsense syllables	We are going to play a game. I am going to say words, but they don’t mean anything. I want you to use the sounds we worked on today and copy me as best you can. (Use nonsense words produced by the Speech Motor Learning (SML) Programme (Van der Merwe, 2011), make use of modelling to correct where needed and positive verbal reinforcement for correct productions).
28–35	Phoneme–grapheme coupling: Reading	I am going to show you 10 cards, one at a time. I would like to you try and read the word before you hear this sound (ring bell). When the bell goes, I will tell you what the word is and then we will go on to the next word.
36–45	Phoneme–grapheme coupling: Spelling	Here I have cards with the different ways we can write it when a sound sounds like this (produce target sound). I am going to say a word and I want you to tell me on which side of the page it goes. Then we will decide what sounds belong in that word and write it together.
